# Ventral striatal dopamine transporter availability is associated with lower trait motor impulsivity in healthy adults

**DOI:** 10.1038/s41398-018-0328-y

**Published:** 2018-12-07

**Authors:** Christopher T. Smith, M. Danica San Juan, Linh C. Dang, Daniel T. Katz, Scott F. Perkins, Leah L. Burgess, Ronald L. Cowan, H. Charles Manning, Michael L. Nickels, Daniel O. Claassen, Gregory R. Samanez-Larkin, David H. Zald

**Affiliations:** 1 Department of Psychology, PMB 407817, Vanderbilt University, 2301 Vanderbilt Place, Nashville, TN 37240-7817 USA; 2 Department of Psychiatry and Behavioral Sciences, Vanderbilt University Medical Center, 1601 23rd Avenue South, Suite 3057, Nashville, TN 37212 USA; 3 Department of Radiology and Radiological Sciences, Vanderbilt University Medical Center, Medical Center North, 1161 21st Avenue South, Nashville, TN 37232 USA; 4 Department of Chemistry, Vanderbilt University, 7330 Stevenson Center, Station B 351822, Nashville, TN 37235 USA; 5 Department of Biomedical Engineering, PMB 351826, Vanderbilt University, 2301 Vanderbilt Place, Nashville, TN 37235-1826 USA; 6 Department of Neurological Surgery, Vanderbilt University Medical Center, 1161 21st Avenue South, T4224 Medical Center North, Nashville, TN 37232-2380 USA; 7 Department of Neurology, Vanderbilt University Medical Center, 1161 21st Avenue South, A-0118, Nashville, TN 37232-2551 USA; 8 Department of Psychology and Neuroscience, Duke University, 417 Chapel Drive, Durham, NC 27708 USA

## Abstract

Impulsivity is a transdiagnostic feature of a range of externalizing psychiatric disorders. Preclinical work links reduced ventral striatal dopamine transporter (DAT) availability with heightened impulsivity and novelty seeking. However, there is a lack of human data investigating the relationship between DAT availability, particularly in subregions of the striatum, and the personality traits of impulsivity and novelty seeking. Here we collected PET measures of DAT availability (BP_ND_) using the tracer ^18^F-FE-PE2I in 47 healthy adult subjects and examined relations between BP_ND_ in striatum, including its subregions: caudate, putamen, and ventral striatum (VS), and trait impulsivity (Barratt Impulsiveness Scale: BIS-11) and novelty seeking (Tridimensional Personality Questionnaire: TPQ-NS), controlling for age and sex. DAT BP_ND_ in each striatal subregion showed nominal negative associations with total BIS-11 but not TPQ-NS. At the subscale level, VS DAT BP_ND_ was significantly associated with BIS-11 motor impulsivity (e.g., taking actions without thinking) after correction for multiple comparisons. VS DAT BP_ND_ explained 13.2% of the variance in motor impulsivity. Our data demonstrate that DAT availability in VS is negatively related to impulsivity and suggest a particular influence of DAT regulation of dopamine signaling in VS on acting without deliberation (BIS motor impulsivity). While needing replication, these data converge with models of ventral striatal functions that emphasize its role as a key interface linking motivation to action.

## Introduction

Impulsivity is a transdiagnostic feature of psychopathology and is a particularly prominent symptom of externalizing disorders, including attention-deficit/hyperactivity disorder (ADHD), conduct disorder, antisocial personality disorder, and substance/alcohol use disorders. The dopamine transporter (DAT), along with the norepinephrine transporter (NET^1^), is a key molecular target for psychostimulants (methylphenidate, (d-)amphetamine) used to treat ADHD^2,3^. These psychostimulants have been shown to reduce impulsive symptomology (e.g., response inhibition as measured by faster stop-signal reaction times^4^; physician’s rating of short attention span and hyperactivity^5^) and increase self-control^6,7^. Given DAT’s critical role in striatal dopamine (DA) function and existing theoretical links between DA and impulsivity, individual differences in DAT are of particular interest as a potential influence on impulsivity. Individual differences in striatal DA signaling have been shown to relate to differences in trait impulsivity in human subjects^8,9^. Specifically, striatal d-amphetamine-induced DA release has been reported to positively correlate with Barratt Impulsiveness Scale (BIS-11^10^) scores in healthy individuals^8^. As d-amphetamine releases DA by blocking reuptake and reversing DAT transport of DA^11,12^, individual differences in responses to d-amphetamine could potentially indicate that DAT levels influence degree of impulsivity. While studies addressing this relationship are scarce, they are critically needed given that understanding DAT’s role in trait impulsivity could provide insights into the etiology and treatment of impulsivity in externalizing disorders. However, among preclinical and human studies that have investigated the relationship between DAT and impulsivity specifically, there remain questions about the direction of the observed relationship given the potential varied functions DAT plays in DA signaling across different timescales and disease states.

The conflicting hypotheses about DAT and impulsivity in part reflects a core issue in the interpretation of measured DAT availability in human positron emission tomography (PET) and single photon emission computed tomography (SPECT) imaging. One potential interpretation is that measured DAT availability provides an index of DA regulatory capacity. DAT is critical in curtailing the large amount of DA released from phasic DA neuron spiking^13^, and thus its availability may indicate the extent to which DA released into the synaptic cleft in response to salient stimuli can be regulated. If DAT is primarily indexing regulatory control, low DAT availability (due to lower surface expression or a greater number of transporters in a lower affinity conformational state) may represent a decreased ability for individuals to limit DA overflow in response to stimulation. Consistent with this model of DAT-binding indexing regulatory capacity, rodents with knockout of DAT show signs of heightened dopaminergic tone as reflected in heightened responses to novelty and spontaneous hyperlocomotion^14,15^. This preclinical work would suggest that lower DAT availability in humans would be associated with hyperactivity and novelty responding, which are often correlated with impulsivity and are key behavioral hallmarks of ADHD. It should also be noted that more modest reductions in DAT have sometimes^16^, but not always, been associated with enhanced impulsivity in rodents^17,18^. This inconsistency could be the result of the particular subdivision of the striatum where DAT reduction has occurred. Among the strongest evidence to date for a regulatory deficit model of lowered ventral striatal (VS) DAT causing impulsivity comes from work by Jupp et al. (2013), who found that rats with stably high impulsive (premature responding) performance on the five-choice serial reaction time task had lower DAT availability in the nucleus accumbens shell but not in the core or dorsal striatum as assessed with autoradiography^19^. Combined with data that high impulsive rats showed enhanced electrically induced DA release in the nucleus accumbens shell^20^, the authors interpret their result as an anatomically specific under-regulation of DA signaling due to impaired clearance of DA.

Another potential model for the interpretation of DAT availability is to treat it as a presynaptic index of overall DA system signaling capacity, reflecting a higher amount of DA neuron terminals. This model is often cited in the Parkinson’s Disease literature, which uses DAT availability as a measure of DA system integrity^21–24^. However, although the positive relationship between striatal DAT and other presynaptic markers of DA system integrity is robust in those with damaged DA neurons due to Parkinson’s or MPTP exposure, it appears much weaker in individuals with intact DA systems or normal levels of DAT^25^, likely reflecting the fact that there are substantial influences on DAT trafficking^26^ and DA synthesis^27^ that are superimposed on individual differences in the number of striatal DA terminals. It is thus unclear to what extent individual differences in DAT availability reflects overall DA system integrity in neurologically healthy adults.

Only two published SPECT studies in healthy adults to date have reported on the relationship between DAT availability and trait impulsivity in humans. While one reported that higher striatal DAT availability was positively correlated with trait impulsivity^28^, this effect was not observed in a large study using a similar tracer^29^. In addition to the relatively low sensitivity and spatial resolution of SPECT imaging^30^, a limitation of both of these studies is that they did not address VS DAT, focusing instead on either whole striatal values or caudate and putamen values. This limitation could be important given Jupp’s^19^ finding that reduced DAT in the nucleus accumbens (but not dorsal striatum) of rats is associated with higher impulsivity. Additionally, D2-like receptor reductions in high impulsive animals have been principally observed in the ventral rather than in the dorsal striatum^31^.

Impulsivity is a heterogeneous trait. For instance, factor analysis of the BIS-11 demonstrates the presence of three different factors (Attentional, Motor, and Nonplanning impulsivity^10^), which have been found frequently to differentially correlate with a variety of psychiatric disorders^32,33^, other measures (behavioral and self-report) of impulsivity^33^, and electrophysiological^34^ and neuroimaging markers^35^. Impulsivity is also closely related to the construct of novelty seeking (NS), which has been also linked to DAT functions. Preclinical work has found that high novelty responding rodents^36^ have decreased DAT affinity and reduced DA uptake at baseline^37^. Furthermore, knockdown of DAT is associated with increased hyperactivity in a novel environment and enhanced exploration of a novel object in mice^38^. In humans, NS is typically measured with the Tridimensional Personality Questionnaire (TPQ-NS)^39^, and heightened TPQ-NS was hypothesized by Cloninger to be the result of low basal DA tone^40,41^. One SPECT study has reported a positive correlation between DAT availability and TPQ-NS scores in predominantly male treatment-seeking alcoholic subjects but not controls^42^. The interpretation of this finding is complicated by the mixture of subjects with widely varying alcohol use histories. In addition, a recent SPECT study in opioid-dependent individuals found an inverted-U-shaped relationship between striatal DAT and TPQ-NS in male patients but a non-significant, positive linear relationship in controls^43^. As with the SPECT studies of impulsivity, no examination of the VS was provided in either NS study.

In summary, there is a discrepancy in results between existing animal and human studies of DAT and impulsivity/NS, with the failure to separately assess the VS in the human SPECT studies possibly contributing to this discrepancy. Here we tested for a relationship between the personality traits of impulsivity and NS and DAT availability assessed with the radiotracer [^18^F]-FE-PE2I ((E)-N-(3-iodoprop-2-enyl)-2beta-carbofluoroethoxy-3beta-(4’-methyl-phenyl)nortropane). FE-PE2I has been used successfully in both non-human primates^44,45^ and human studies^46,47^ and has excellent reproducibility of nondisplaceable binding potential (BP_ND_) estimates in striatum^48^. With the higher spatial resolution afforded by PET, we focused our FE-PE2I analysis on striatal subdivisions (caudate, putamen, and ventral striatum), which extends previous SPECT work that did not examine VS DAT^28,29,42,43^. While the past SPECT studies might suggest an opposing hypothesis, given previous preclinical results^19,37,38^, we predicted a negative relationship between VS DAT and aspects of impulsivity/NS in healthy human subjects.

## Methods

### Participants and procedure

Forty-seven healthy participants (in two cohorts of 20–30-year olds (*n* = 23, 13 male) and 50–65-year olds (*n* = 24, 11 male), mean = 41.1 ± 15.5) underwent an [18 F]-FE-PE2I PET scan as part of a larger ongoing study. Participants were recruited from the Nashville, TN area and all participants completed written informed consent approved by the Vanderbilt University Institutional Review Board. Exclusion criteria included any axis-1 psychiatric disorder, use of psychoactive drugs, illicit drug use, and alcohol consumption ~>5 standard alcoholic drinks/week. All participants underwent a structured clinical interview^49^ for these exclusionary criteria in addition to a medical physical (with electrocardiogram), a complete blood count panel, and structural magnetic resonance (MR) scans (T1 and T2-FLAIR weighted) to exclude pathology. All participants in the study passed a urine drug screen (Construction 12-Drug Screen Test; Innovacon, Inc., San Diego, CA) for commonly abused drugs, including THC, cocaine, amphetamine, opiates, barbiturates, and benzodiazepines, administered on a separate prescreening/physical examination day.

### Trait impulsivity and NS measures

Participants completed the BIS-11 and TPQ-NS questionnaires and total and subscale scores for each were calculated, including the 3-factor subscale solution for BIS-11^10^ and 4 subscales for TPQ-NS^39^.

### PET imaging

[^18^F]-FE-PE2I was produced by the Vanderbilt Radiochemistry Core laboratory under cGMP protocols. The synthetic preparation was carried out utilizing a Tracerlab FXFN reaction module (GE Healthcare) and was synonymous to a previously published preparation^50^. Serial scan acquisition was started simultaneously with a slow bolus injection (mean = 4.63 ± 0.34 mCi; 171.31 ± 12.58 MBq) of the DAT tracer [^18^F]-FE-PE2I (specific activity = 2302 ± 1445 Ci/mmol). CT scans were collected for attenuation correction prior to the emission scan. FE-PE2I data were collected during one 61-min dynamic scan with acquisition times listed in Table S1. While previous human PET studies using FE-PE2I used 90 min dynamic scan acquisitions^46,48^, we chose the 61 min acquisition time to improve participant comfort given that the correlation between putamen FE-PE2I BP_ND_ collected after 61 and 90 min in 4 pilot subjects was extremely high (*r* = 0.997, mean percentage of BP_ND_ difference = 2.06 ± 1.72). All PET data were collected on a GE Discovery STE PET scanner (GE Healthcare, Milwaukee, USA).

### PET data processing

After decay correction and attenuation correction, PET scan frames were corrected for motion using SPM8^51^ with the 20th frame of dynamic scan series serving as the reference image. The mean PET image created from the realignment was then registered to each subject’s high-resolution T1 MR image (FLIRT, 6 degrees of freedom), which was nonlinearly registered to MNI space (FNIRT) in FSL^52^. Putamen and cerebellum reference regions of interest (ROIs) were created from the WFU Pickatlas^53^ with the cerebellum modified such that the anterior ¼ of the ROI along with voxels within 5 mm of cortex were excluded to prevent contamination of the PET signal from nearby areas, such as midbrain or occipital cortex. These ROIs were then warped to each subject’s PET space using the FLIRT and FNIRT FSL transform matrices (MNI → T1 → PET) and used in a simplified reference tissue model (SRTM^54^) performed in the PMOD software (PMOD Technologies, Zurich Switzerland) to estimate FE-PE2I BP (BP_ND_, a ratio of specifically bound FE-PE2I to its non-displaceable concentration). Specifically, PMOD’s PXMOD tool was used to estimate BP_ND_ voxelwise using a published basis function fitting approach^55^. Previous work has demonstrated FE-PE2I SRTM BP_ND_ in healthy participants is highly reproducible in striatum^48^; intraclass correlation coefficients: 0.83 in caudate, 0.88 in putamen.

Because we were particularly interested in DAT BP_ND_ in basal ganglia, we performed all analyses using subject-specific PET space ROIs. Template caudate, putamen, and ventral striatum (VS) ROIs as defined in Mawlawi et al.^56^ were warped from MNI space to each subject’s PET space in a manner (MNI → T1 → PET) similar to the cerebellum and putamen ROIs used in the estimation of BP_ND_. Caudate, putamen, and VS PET space ROIs were thresholded at 90% probability and then mean BP_ND_ values were extracted for each participant in FSL. In addition, subject-specific BP_ND_ images were also warped to MNI space using the saved FSL transforms to create MNI-normalized BP_ND_ images (resampled to 2 mm isotropic voxels) for use in exploratory voxelwise analyses. These voxelwise analyses were implemented as multiple regressions in SPM8, controlling for participant age and sex. All analyses used an explicit mask of the striatum as FE-PE2I signal was most robust in this region of the brain (see Supplementary Figure 1). Criteria for significance in the SPM8 analyses was set at a *p* < 0.005 threshold and required meeting a false discovery rate (FDR) or familywise error rate (FWE) correction for multiple comparisons at either the cluster or peak level of *p* < 0.05.

### Statistical analyses

**Fig. 1:**
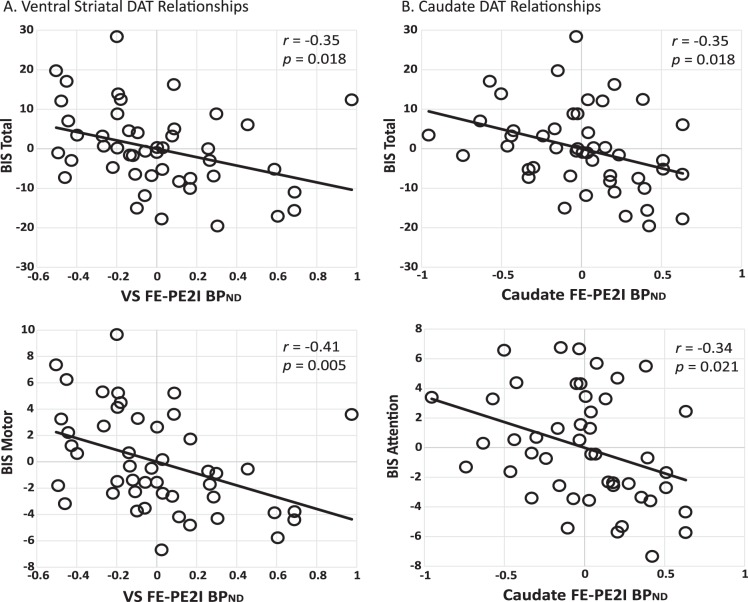
Partial correlation plots of significant relationships between striatal FE-PE2I BP_ND_ and impulsivity. Partial correlation plots from the regression analyses, controlling for age and sex, are displayed for the striatal regions whose FE-PE2I BP_ND_ (DAT availability) were most predictive of BIS-11 scores. **a** Ventral striatum relationships with BIS-11 total (95% CI for *r*: −0.64, −0.05) and BIS Motor subscale (95% CI for *r*: −0.65, −0.13). **b** Caudate relationships with BIS-11 total (95% CI for *r*: −0.56, −0.12) and BIS Attention subscale (95% CI for *r*: −0.57, −0.097). Note that Caudate BP_ND_ was slightly more predictive of BIS-11 total than ventral striatum but the effect sizes were similar. The ventral striatal correlation with BIS Motor subscale was significant at Bonferroni correction of *p* < 0.0056. BIS Barratt Impulsiveness Scale, VS ventral striatum

First, we tested for a relationship between FE-PE2I BP_ND_ in our three striatal ROIs using correlation analyses. We then tested for predictors of BIS-11 total score via multiple regression analyses with age and sex entered in the first level and striatal FE-PE2I BP_ND_ from any of the three ROIs allowed to be entered stepwise in the second level. We also report partial correlation results with 95% confidence intervals (CIs) to indicate the strength of our observed effects (see Fig. 1), controlling for age and sex, as they can affect DA signaling^57–60^. If an ROI showed at least a nominally significant association with total BIS-11, we performed follow-up multiple regression analyses (with correction for multiple comparisons) to test for relationships between FE-PE2I BP_ND_ and each of the BIS-11 3-factor^10^ subscales (Attentional, Motor, and Nonplanning impulsivity), in order to see if a specific aspect of impulsivity was associated with regional DAT. We applied a similar strategy for the analysis of TPQ-NS. Raw BP_ND_, BIS-11, and TPQ-NS data along with the partial correlation data used to construct Fig. 1 can be obtained from the Open Science Framework website at https://osf.io/324tw/. Finally, given that DAT availability declines with age^61^, we also examined whether there was an interaction between age and DAT in predicting personality traits.

## Results

### Personality measures

BIS-11 and TPQ-NS scores for the sample as a whole and for both age groups are reported in Table 1. BIS-11 and TPQ-NS scores were not significantly different by age group. There was also no effect of sex on the personality measures (max. *T* = 1.73, min. *p* = 0.091 (females higher) for BIS Nonplanning subscale; *T* = 1.59, *p* = 0.12 for BIS-11 Total; *T* = 1.00, *p* = 0.32 for TPQ-NS Total).

**Table 1:** Comparison of age groups on variables of interest

Age group	Full group (*n* = 47)	Young adults (*n* = 23)	Middle-aged adults (*n* = 24)	*T*, *p*
	Mean (SD)	Mean (SD)	Mean (SD)	
Age	41.11 (15.47)	25.78 (2.61)	55.79 (3.53)	−33.05, <0.001
Sex (% male)	51.1	56.5	45.8	0.54, 0.46^a^
FE-PE2I VS BP_ND_	2.29 (0.48)	2.62 (0.45)	1.98 (0.25)	6.05, <0.001
FE-PE2I caudate BP_ND_	2.02 (0.54)	2.40 (0.40)	1.65 (0.41)	6.30, <0.001
FE-PE2I putamen BP_ND_	3.26 (0.44)	3.54 (0.40)	2.99 (0.27)	5.57, <0.001
BIS Total	58.28 (10.74)	57.30 (9.37)	59.21 (12.04)	−0.60, 0.55
BIS Motor	21.57 (3.80)	21.13 (3.57)	22.00 (4.04)	−0.78, 0.44
BIS Attention	15.49 (3.85)	15.65 (3.42)	15.33 (4.29)	0.28, 0.78
BIS Nonplanning	21.21 (4.68)	20.52 (4.13)	21.88 (5.15)	−0.99, 0.33
TPQ-NS Total	13.26 (5.00)	14.09 (3.85)	12.46 (5.88)	1.13, 0.27
NS1	5.06 (1.97)	5.26 (1.79)	4.88 (2.15)	0.67, 0.51
NS2	2.11 (1.89)	2.00 (1.83)	2.21 (1.98)	−0.37, 0.71
NS3	2.62 (1.42)	2.87 (1.25)	2.38 (1.56)	1.20, 0.24
NS4	3.47 (1.73)	3.96 (1.55)	3.00 (1.79)	1.95, 0.57

We found significant differences in FE-PE2I BP_ND_ between age groups. There was no difference in sex distribution or in BIS-11 and TPQ-NS Scores across age groups

*SD* standard deviation

^a^Result of Chi-Square test (df = 1)

### FE-PE2I BP_ND_ across striatal subregions

FE-PE2I SRTM BP_ND_ across the three striatal subdivisions are reported for the total sample and for each age group in Table 1. We note that, using the present methods, BP_ND_ estimates are lower than those reported previously in human subjects^46,48^. Nevertheless, the pattern of results is similar to past studies, as previous FE-PE2I PET studies have similarly reported higher SRTM BP_ND_ in putamen relative to caudate^46,48^ and immunocytochemical localization of DAT protein in postmortem human brain has been found to be qualitatively higher in putamen than in caudate and ventral striatum^62^. FE-PE2I BP_ND_ was also highly correlated across the striatal ROIs with *r* values >0.7: caudate to putamen (*r* *=* 0.738, *p* < 0.001; CI: 0.578, 0.857), caudate to VS (*r* *=* 0.736, *p* < 0.001; CI: 0.621, 0.844), and putamen to VS (*r* *=* 0.730, *p* < 0.001; CI: 0.580, 0.869), as expected. Also, we found that the middle-aged adult cohort, as expected^47^, had lower FE-PE2I BP_ND_ than young adults in caudate, putamen, and VS (all *p* < 0.001, see Table 1).

### Relationship between personality and FE-PE2I BP_ND_

We found that total BIS-11 scores were almost equally as well predicted by FE-PE2I BP_ND_ in caudate (*β* = −0.505, *F*-change from age and sex model: *F* = 6.10, *p* = 0.018, total model *R*^2^ = 0.121) and VS (*β* = −0.477, *F*-change from age and sex model: *F* = 6.06, *p* = 0.018, total model *R*^2^ = 0.120) singularly. Putamen FE-PE2I BP_ND_ was less strongly predictive of BIS-11 (*β* = −0.384, *p* = 0.048) but followed the same relationship as caudate and VS. The addition of FE-PE2I BP_ND_ from no other striatal region in combination with either caudate or VS added additional prediction to the model, most likely due to the high correlation between BP_ND_ between the striatal areas (*r*s ≈ 0.73, see above). While the relationship between BIS-11 total scores and FE-PE2I BP_ND_ in caudate and VS explained (with age and sex) ~12% of the variance in BIS-11 score, the *p* values of the relationships did not survive correction for multiple comparisons using the most stringent correction criteria, Bonferroni (*p* < 0.0167).

Given the preclinical literature that also suggests that aspects of impulsivity are inversely related to DAT^19,31,38^ and human studies demonstrating differential relationships between BIS-11 subscales and a variety of impulsive behaviors^33^ and neural measures^34,35^, we next investigated whether there were any subscale-specific associations with FE-PE2I BP_ND_. We tested for the ability of VS, caudate, and putamen FE-PE2I BP_ND_ to predict BIS-11 subscale scores, with our critical *p* value set at 0.0056 (Bonferroni correction for 9 tests: 3 BIS subscales × 3 ROIs). At the subscale level, BIS Motor subscale scores were best predicted by VS FE-PE2I BP_ND_ (*β* = −0.568, *F*-change from age and sex model: *F* = 8.70, *p* = 0.005, total model *R*^2^ = 0.132) and the relationship between these two measures survived Bonferroni correction. BIS Attention subscale was best predicted by caudate FE-PE2I BP_ND_ (*β* = −0.491, *F*-change from age and sex model: *F* = 5.73, *p* = 0.021, total model *R*^2^ = 0.113) but did not survive Bonferroni correction. Nonplanning subscale was not significantly predicted by FE-PE2I BP_ND_ in any region. In no case did FE-PE2I BP_ND_ from multiple striatal regions better predict BIS-11 subscale scores than the one observed region (VS and Motor subscale, caudate and Attention subscale). Figure 1 displays scatter plots of the partial correlation results (controlling for participant age and sex) showing FE-PE2I BP_ND_ from the specific striatal region that was most predictive of BIS-11 total or subscale scores.

Given that the size of striatal regions could influence BP_ND_ estimates and striatal subregions have shown associations with aspects of impulsivity in some studies^63–65^, we additionally examined associations between gray matter volume and impulsivity. However, we observed no significant associations, indicating that the observed findings between FE-PE2I BP_ND_ and impulsivity were not a result of differences in gray matter volume (see Supplemental Materials).

Voxelwise analyses conducted in SPM8 on the FE-PE2I BP_ND_ images warped to MNI space confirmed the negative relationship between BIS-11 scores and FE-PE2I BP_ND_ in caudate and VS, though the clusters did not reach significance after FDR or FWE correction (unthresholded statistical maps available on Neurovault: https://neurovault.org/collections/3911/).

FE-PE2I BP_ND_ from no striatal region significantly predicted total TPQ-NS scores (max. *β* = −0.37, min. *p* = 0.069 for putamen BP_ND_).

### Does age modulate the relationship between DAT and BIS-11?

The present study included both young and middle-aged participants, under the assumption that DAT levels have an impact on impulsivity across a broad age range. While we controlled for age in all analyses involving PET data, given the significant age effect on FE-PE2I BP_ND_ it is possible that aging impacts the relationship between DAT and impulsivity. Thus we tested for an interaction between age group and FE-PE2I BP_ND_ (*Z*-standardized within age groups) on BIS-11 in each striatal subdivision. We found no significant interaction in caudate (*F*_(1,43)_ = 2.59, *p* = 0.115), putamen (*F*_(1,43)_ = 0.30, *p* = 0.590), or VS (*F*_(1,43)_ = 1.39, *p* = 0.244). We also found no age group×VS FE-PE2I BP_ND_ interaction on BIS Motor Impulsivity (*F*_(1,43)_ = 0.51, *p* = 0.479).

## Discussion

In this study, we investigated whether striatal DAT availability as measured by FE-PE2I BP_ND_ was predictive of trait impulsivity (BIS-11) in healthy adults. We specifically focused on striatal subregions (caudate, putamen, and VS) given strong preclinical work suggesting a negative relationship between VS DAT and impulsive behavior. In a sample of 47 healthy adults, we found that caudate and VS FE-PE2I BP_ND_ explained variance in BIS-11 Total scores such that lower FE-PE2I was nominally associated with higher BIS-11 scores. When analyzed at the subscale level, VS FE-PE2I BP_ND_ was particularly associated with motor impulsivity, which reflects taking action without first thinking about the potential consequences of the action. The strong inverse relationship between VS DAT and motor impulsivity fits with preclinical data, demonstrating that reduced DAT in this region relates to impulsive premature responding^19^ and that the VS serves as a limbic–motor interface where affective/internal state modulates the readiness to perform behavioral actions^66^.

### Lower DAT, reduced DA regulatory capacity, and heightened impulsivity

We interpret the link between lowered VS DAT availability (FE-PE2I BP_ND_) and heightened motor impulsivity as reduced regulatory capacity in impulsive individuals. DAT is critical in curtailing the magnitude and duration of DA signaling induced by phasic DA neuron spiking^13,67^ and limits DA spillover outside the synapse^68^. There is also work showing that DAT plays a role in limiting the diffusion of DA to high-affinity D2 receptors located at some distance (~10 μm) from release sites in striatum^69,70^ and could presumably affect the degree to which D2 autoreceptors (which normally control DA signaling) are stimulated by extracellular DA. As such, DAT appears critical in determining the level of regulation over DA signaling in response to salient stimuli that causes phasic DA release^71^. If this regulatory model for DAT is applied to our data, low DAT availability in VS may represent a decreased ability for highly impulsive individuals to constrain DA signaling in response to stimulation. This interpretation fits with previous findings that individuals presumed to have less regulation of DA due to decreased midbrain autoreceptor D2/3 availability show greater impulsivity^8,72^.

### Caveats in interpreting DAT availability measure

It is important to note, however, that DAT availability assessed with PET or SPECT indexes a combination of: (1) number of presynaptic DA terminals, and (2) the amount of DAT protein expressed on the surface membranes of DA terminals available to bind the tracer. Given that our subjects were neurologically healthy individuals whose DA system is presumed to be intact, we emphasized the second component (the amount of DAT protein per terminal) in our interpretation of the data. Nevertheless, we cannot say whether reduced DAT availability primarily indexes decreases in DA terminal number or decreases in DAT expression. Thus reduced DAT availability may also be indexing lower DA terminal number and thus reduced overall DA signaling capacity in individuals with higher levels of trait impulsivity. Future combined microPET and immunochemical microscopic work with animals would be informative in clarifying the extent of FE-PE2I PET signal attributable to DAT protein number versus DA terminal number. We also note that differences in the conformational state of expressed DAT could contribute to its regulatory ability by influencing uptake of DA and its regulation^73^, but data on the extent to which this might contribute to FE-PE2I is not currently known.

### Comparing current findings with the existing data

Past preclinical data suggest that lowered DAT functions may lead to heightened valuation of novel rewarding stimuli^74^. However, in the present study, we did not observe a strong correlation between TPQ-NS and striatal DAT availability. If trying to identify the specific personality correlates of DAT levels, the present data suggest that trait features of impulsivity rather than characteristics specific to NS are most related to DAT availability.

Our results diverge from two previous SPECT studies that investigated the relationship between DAT availability and impulsivity^28,29^, with one finding a positive relationship between caudate and putamen DAT availability and BIS-11 in healthy male subjects^28^ while another detected no relationship despite the use of a similar radiotracer^29^. Neither SPECT study explicitly assessed VS DAT despite preclinical work^19^ suggesting its specific involvement with impulsivity. We note that the study demonstrating a positive relationship between BIS and striatal DAT used a German version of the BIS that, while internally consistent and reliable, does not capture the same factor structure as the English BIS-11^75,76^. That study also included the personality trait of neuroticism as a control variable and differed from the present study in its restriction to only males^28^. The other SPECT study, which failed to observe significant associations, also differs from the current study in that it examined a population who had significant drug use histories. Although subjects had to abstain from drug use for the 2 weeks prior to scanning, they were explicitly recruited because they indicated a high probability of trying the drug Ecstasy in the near future^29^.

An additional source of the discrepant results across studies may arise from the specific properties of the DAT ligands employed. [123I]β-CIT used most prominently in SPECT imaging of DAT has high affinity for the serotonin transporter (SERT)^77^ and is based off a chemistry sensitive to selective serotonin reuptake inhibitor medications^78,79^. In fact, FP-CIT tracers have been used to image SERT^80,81^, which has reasonably high distribution in the striatum^82,83^. The previously observed positive relationship between [123I]FP-CIT BP_ND_ and BIS-11^28^ could thus result from differences in FP-CIT specificity for DAT relative to SERT versus the greater DAT specificity of the FE-PE2I tracer we used here^84^. Despite this concern, work in non-human primates suggests that FP-CIT signal in striatum is relatively specific for DAT over SERT^85^. More work is needed to dissociate the relative signals of the monoamine transporters using PET/SPECT radiotracers and new tracers may solve some of these issues^86,87^.

### DAT, aging, and impulsivity

A challenge in studying DA and psychological phenotypes is that there are robust declines in many aspects of the DA system with age^61^. Our finding of a decline in striatal DAT with age is consistent with a past PET study using FE-PE2I^47^. Interestingly, despite a clear decline in striatal DAT with age, the relationship between DAT and personality across individuals was consistent across age groups after accounting for the effect of age. This suggests that trait relationships with DAT are maintained at least into late middle age despite the changes in the dopaminergic system across adulthood.

### Implications of our findings for ADHD

If, as we interpret it, measured DAT in healthy young and middle-aged adults is primarily indexing DA regulation capacity, it may seem surprising that we found an inverse relationship between VS DAT availability and motor impulsivity given that psychostimulant treatments that block DAT reduce impulsivity^4^ and increase self-control^6,7^. It should be noted, however, that, while DAT is a target for psychostimulants (such as methylphenidate, d-amphetamine) used to treat ADHD, they also target NET and SERT^88^. Thus the therapeutic efficacy of psychostimulants on impulsivity may not be at DAT, and thus DAT’s relationship to impulsivity may be independent of the effects of methylphenidate and/or (d-)amphetamine on ADHD symptoms. Studies investigating the relationship between DAT availability and ADHD using PET/SPECT have been inconsistent, with a meta-analysis showing that ADHD medication history may explain this inconsistency^89^: drug-naive ADHD patients tend to have lower DAT availability compared to controls while ADHD patients treated with psychostimulants display higher DAT availability compared to controls. Indeed, sustained methylphenidate treatment, which reduces ADHD symptomology (response inhibition^4^; inattention and hyperactivity^5^), has been shown to increase DAT availability^90^. Importantly, the largest PET study in the meta-analysis that excluded ADHD individuals with past psychostimulant treatment found *lower* DAT availability in the striatum of ADHD relative to control individuals^91^. Thus our observation of lower DAT availability being associated with higher trait impulsivity fits with lower DAT observed in treatment-naive individuals with ADHD. Additionally, our work suggests that particular components of impulsivity in ADHD might be associated with DAT levels in specific striatal subregions: with VS contributing more to motor impulsivity (i.e. acting on impulse) and the caudate showing somewhat greater association with attentional impulsivity (i.e., difficulty concentrating). However, at least in the present data, this does not appear to be a true double dissociation, which is not entirely surprising given the observed correlations between DAT expression in the striatal regions, and the correlations of the different BIS-11 subscales with each other. Nevertheless, future work should investigate whether the modulation of DAT signaling in specific striatal subregions results in different effects on the motor versus attentional aspects of impulsivity in the context of ADHD and other externalizing phenotypes. If the relationships we observed are confirmed in more detailed preclinical behavioral models, this work may aid in the development of novel treatments for these particular subcomponents of ADHD.

### Relationships between FE-PE2I BPND, BIS-11, and gray matter volume in striatum/VTA

We conducted exploratory analyses between gray matter volume of striatum and ventral tegmental area (VTA), FE-PE2I BP_ND_, and BIS-11 scores (see Supplemental Materials and Table S2). Previous research has found relationships between striatal gray matter volume and behavioral^63,64^ and trait^92^ impulsivity in human subjects. We did not find a relationship between BIS-11 scores and striatal gray matter volume in our data. The literature on gray matter volume and impulsivity is not consistent with two studies finding a negative relationship between striatal volume and trait impulsivity^63,92^, albeit in different subregions and using different personality scales, and another showing a positive correlation between caudate volume and behavioral impulsivity (from a temporal discounting task)^64^. The challenge of the structural literature is that there are multiple measures being used to assess different aspects of impulsivity, and this has at times limited the ability to replicate findings across studies and species. The present analyses should not be considered a direct attempt to replicate past findings but do suggest that differences in striatal or VTA gray matter are not driving our observed relationship between striatal FE-PE2I BP_ND_ and BIS-11 scores.

### Limitations

While our sample size is larger than many PET studies, our findings of an inverse relationship between DAT availability and trait impulsivity will require replication. In addition, our emphasis on DAT’s role as a regulator of synaptic DA signaling warrants the caveat that DAT may be more important in regulating volume transmission of DA than its synaptic signaling per se^69,93^. Low DAT availability in this volume transmission model would result in DA signaling across a wider brain circuit than would otherwise be the case. This less focused DA transmission would presumably produce more off-target effects as a result of the wider spatial distribution of DA signaling and could affect overall behavioral patterns in distinct ways^94^, perhaps by modifying the signal to noise of relevant action-value signals in striatum^95^. These effects might then manifest as motor impulsivity and lead to the same general finding of lower DAT being associated with heightened trait impulsivity. While we propose a regulatory capacity explanation for DAT’s effects on trait impulsivity, the lack of published data in vivo in nonhuman primates makes it difficult to confidently infer a clear mechanistic explanation for the relationship between DAT availability, DA signaling, and impulsivity in humans.

In addition, while we found relationships between trait impulsivity and DAT, our range of impulsivity was limited in this sample of psychiatrically healthy adults. This could have limited our detection of relationships. It is possible, for instance, that the caudate DAT relationship with BIS Attention and Nonplanning impulsivity would have been stronger if the range in these scores were larger. As this study focused on healthy adults, our findings may also be limited in their generalizability to patient populations, including those with ADHD. Investigating relationships between particular facets of impulsivity and DAT availability in clinical populations is needed.

## Conclusion

We observed an inverse relationship between striatal DAT availability and trait impulsivity in healthy young and middle-aged adults. Motor impulsivity showed a statistically significant relation with VS DAT after correction for multiple comparisons, suggesting potential regional and phenotypic specificity. The findings are consistent with preclinical data and have implications for the role of DA signaling in the impulsive traits observed in externalizing disorders. Future work that integrates DAT with other measures of DA signaling dynamics should allow for a more complete understanding of the role of DA functioning in impulsivity.

## Supplementary Information

Supplementary Material
