# Supplementary material for: Ventral striatal dopamine transporter availability is associated with lower trait motor impulsivity in healthy adults
Source: Transl Psychiatry. 2018 Dec 7;8:269. doi: 10.1038/s41398-018-0328-y (PMC6286354; doi:10.1038/s41398-018-0328-y)
Supplement: Supplementary file 1 — Supplementary Material [file 41398_2018_328_MOESM1_ESM.docx]

**Ventral Striatal Dopamine Transporter Availability is Associated with Lower Trait Motor Impulsivity in Healthy Adults**

Smith et al.

**Supplementary Analyses**

*Relationship between mesolimbic dopamine system gray matter volume and FE-PE2I BP*_ND_

Relationships have previously been reported between striatal gray matter volume and behavioral^1,2^ as well as trait^3^ impulsivity in human subjects. Thus, it is possible that gray matter volume of the ventral striatum or the ventral tegmental area (VTA) that projects to it could be driving our observed relationship between FE-PE2I BP_ND_ and BIS-11. This may be especially true if DAT availability indexed by FE-PE2I BP_ND_ reflects overall DA system integrity (the alternative hypothesis to the regulatory hypothesis we propose for our observed DAT effect). If that was the case, one might expect the gray matter volume in the ventral striatum or VTA (a rough index of DA neuron numbers/density) to relate positively to DAT availability. We tested for these possibilities.

We used FSL FIRST^4^ to segment the striatum in our participants, obtained gray matter volume (mm^3^) for the FIRST caudate, putamen, and VS outputs, and converted them into ROIs for extraction of FE-PE2I BP_ND_ values in our BP_ND_ maps registered to structural MRI (T1) space.

Importantly, we found no relationship between any striatal ROI volume and BIS-11 total or subscale scores (max *r*=-0.24, min *p*=0.11 for BIS Nonplanning and Putamen Vol correlation, Supplementary Table 2). The relationship between VS volume and BIS-11 total score (*r*=-0.011, *p*=0.94) and BIS Motor subscale (*r*=0.11, *p*=0.45) were minimal.

In addition, the relationship between VS volume and FE-PE2I BP_ND_ (*r*=-0.13, *p*=0.38) did not support the interpretation that FE-PE2I BP_ND_ is indexing mesolimbic DA system integrity as expressed in the size of VS.

Finally, to further explore whether VS FE-PE2I BP_ND_ was related to overall DA system integrity, we traced the VTA in co-registered T1 and T2-FLAIR-weighted images for these subjects as per guidelines from Murty et al.^5^ and extracted its volume in mm^3^. There was no significant relationship between VTA volume and VS FE-PE2I BP_ND_ (*r*=0.26, *p*=0.082). Furthermore, there was no relationship between VTA volume and BIS-11 total (*r*=-0.10, *p*=0.49) or any of its subscales (max *r*=-0.21, min *p*=0.15 for BIS Nonplanning).

In summary, these additional analyses lend support to our measure of VS FE-PE2I BP_ND_ not being a simple measure of overall DA system integrity and suggests our interpretation of the relationship between low DAT availability and high trait impulsivity as due to reduced DA regulatory capacity is plausible, if needing further study and validation.

**References**

1. Cho SS, Pellecchia G, Aminian K, Ray N, Segura B, Obeso I, et al. Morphometric correlation of impulsivity in medial prefrontal cortex. Brain Topogr 2013; 26(3): 479-487.

2. Tschernegg M, Pletzer B, Schwartenbeck P, Ludersdorfer P, Hoffmann U, Kronbichler M. Impulsivity relates to striatal gray matter volumes in humans: evidence from a delay discounting paradigm. Frontiers in human neuroscience 2015; 9: 384.

3. Caravaggio F, Plitman E, Chung JK, Gerretsen P, Kim J, Iwata Y, et al. Trait impulsiveness is related to smaller post-commissural putamen volumes in males but not females. The European journal of neuroscience 2017; 46(7): 2253-2264.

4. Patenaude B, Smith SM, Kennedy DN, Jenkinson M. A Bayesian model of shape and appearance for subcortical brain segmentation. NeuroImage 2011; 56(3): 907-922.

5. Murty VP, Shermohammed M, Smith DV, Carter RM, Huettel SA, Adcock RA. Resting state networks distinguish human ventral tegmental area from substantia nigra. NeuroImage 2014; 100: 580-589.

**Supplementary Tables & Figures**

| **PE2I PET Frame**  **Acquisition Times (secs)** |
| --- |
| 0-15 |
| 15-30 |
| 30-45 |
| 45-60 |
| 60-75 |
| 75-90 |
| 90-105 |
| 105-120 |
| 120-150 |
| 150-180 |
| 180-210 |
| 210-240 |
| 240-270 |
| 270-300 |
| 300-360 |
| 360-420 |
| 420-480 |
| 480-540 |
| 540-600 |
| 600-660 |
| 660-810 |
| 810-960 |
| 960-1260 |
| 1260-1560 |
| 1560-2010 |
| 2010-2460 |
| 2460-3060 |
| 3060-3660 |

**Table S1. Acquisition times for PE2I PET data.**

| **Striatal Region**  mean (SD) volume in mm^3^ | **BIS-11 Total**  ***r, p*** | **BIS Attention**  ***r, p*** | **BIS Motor**  ***r, p*** | **BIS Nonplanning**  ***r, p*** |
| --- | --- | --- | --- | --- |
| **Caudate**  7295 (1165) mm^3^ | -0.127, 0.395 | -0.126, 0.398 | 0.017, 0.908 | -0.202, 0.174 |
| **Putamen**  9425 (1054) mm^3^ | -0.137, 0.358 | -0.175, 0.241 | 0.083, 0.580 | -0.238, 0.107 |
| **Ventral Striatum**  891 (207) mm^3^ | -0.011, 0.941 | -0.106, 0.477 | 0.113, 0.451 | -0.030, 0.844 |

**Table S2. Correlation table between BIS-11 total and subscale scores and volume of striatal regions of interest.** We correlated FIRST caudate, putamen, and ventral striatum gray matter volume measures against BIS-11 scores, finding no significant relationships.

**
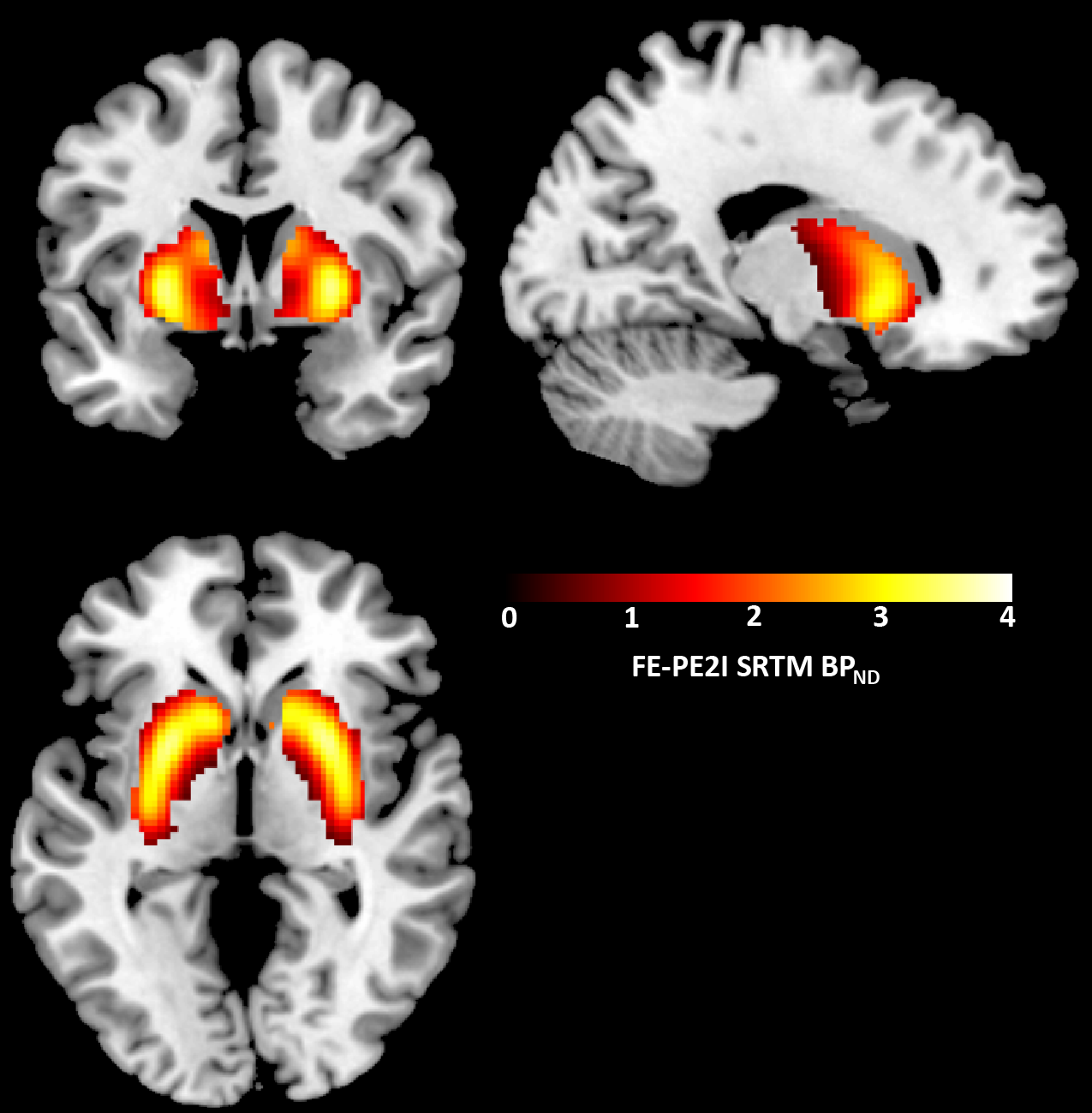
**

**Figure S1. Average FE-PE2I BP_ND_ map from the full dataset.**

Mean FE-PE2I SRTM BP_ND_ for the 47 healthy adults analyzed is displayed in MNI space. Coordinates: 17, 4, -2. Data masked to show signal in brain and remove that found in skull given that dosimetry work (Lizana et al., 2018) shows significant uptake of FE-PE2I in red bone marrow and this is of no interest to the present set of analyses.

Lizana H, Johansson L, Axelsson JE, Larsson Stromvall A, Ogren M, Linder J, et al (2018). Whole-body biodistribution and dosimetry of the dopamine transporter radioligand (18)F-FE-PE2I in human subjects. J Nucl Med.
